# Growth of *Streptococcus mutans* in Biofilms Alters Peptide Signaling at the Sub-population Level

**DOI:** 10.3389/fmicb.2016.01075

**Published:** 2016-07-15

**Authors:** Robert C. Shields, Robert A. Burne

**Affiliations:** Department of Oral Biology, College of Dentistry, University of Florida, GainesvilleFL, USA

**Keywords:** genetic competence, biofilm, ComRS, RelA, type II toxins, quorum sensing

## Abstract

*Streptococcus mutans* activates multiple cellular processes in response to the formation of a complex between *comX*-inducing peptide (XIP) and the ComR transcriptional regulator. Bulk phase and microfluidic experiments previously revealed that ComR-dependent activation of *comX* is altered by pH and by carbohydrate source. Biofilm formation is a major factor in bacterial survival and virulence in the oral cavity. Here, we sought to determine the response of *S. mutans* biofilm cells to XIP during different stages of biofilm maturation. Using flow cytometry and confocal microscopy, we showed that exogenous addition of XIP to early biofilms resulted in robust *comX* activation. However, as the biofilms matured, increasing amounts of XIP were required to activate *comX* expression. Single-cell analysis demonstrated that the entire population was responding to XIP with activation of *comX* in early biofilms, but only a sub-population was responding in mature biofilms. The sub-population response of mature biofilms was retained when the cells were dispersed and then treated with XIP. The proportion and intensity of the bi-modal response of mature biofilm cells was altered in mutants lacking the Type II toxins MazF and RelE, or in a strain lacking the (p)ppGpp synthase/hydrolase RelA. Thus, competence signaling is markedly altered in cells growing in mature biofilms, and pathways that control cell death and growth/survival decisions modulate activation of *comX* expression in these sessile populations.

## Introduction

*Streptococcus mutans* is a principal microorganism contributing to the ubiquitous oral infectious disease dental caries (Loesche, 1986). Recent improvements in DNA sequencing platforms and intensified analysis of the oral microbiome have supported the ecological plaque hypothesis that describes the etiology of the development of the most common oral infectious diseases (Marsh, 1994; Takahashi and Nyvad, 2011). More specifically, perturbations of the environment by the diet, host factors, and endogenous activities of oral biofilms induce changes in microbial composition and behaviors that foster the development of oral infectious diseases. The initiation and progression of dental caries in particular is associated with increases in the proportions of acid tolerant, acid-producing bacteria that rapidly metabolize carbohydrates, leading to repeated acidification of oral biofilms. The acidic environment thus created demineralizes the tooth while concurrently selecting for organisms that are better adapted to growth at low pH. Biofilm formation, growth and metabolism of carbohydrates at low pH, and the ability to respond rapidly to fluctuations in carbohydrate source and availability are attributes of *S. mutans* that are essential for its contributions to the initiation and progression of dental caries (Lemos et al., 2013).

Bacteria use a diverse array of signaling molecules, both intracellular and extracellular, to alter phenotypes in response to changes in their environment effected by host factors, microbial interactions, and exogenously supplied nutrients and chemicals. Second messenger systems that use intracellular signaling molecules have been found to be important governors of bacterial fitness and virulence. Some examples are (p)ppGpp, the primary regulators of the stringent response (Lemos et al., 2007), and cyclic-di-AMP (c-di-AMP), a signal molecule that was recently discovered to influence biofilm formation by *S. mutans* (Peng et al., 2016). Extracellular quorum sensing (QS) molecules that facilitate intra- and inter-species communication are another class of signaling molecules. These include the peptide pheromones of Gram-positive bacteria (Cook and Federle, 2014) and the homoserine lactones of Gram-negative bacteria (Fuqua and Greenberg, 2002). In most cases in nature, bacteria accumulate on surfaces within an extracellular matrix in a community that is generally referred to as a biofilm. Individual organisms in biofilms can have highly variable phenotypes that appear attributable in part to mass transport limitations that create spatial heterogeneity in the concentrations of a variety of molecules, including signaling molecules. Thus, microenvironments within biofilms create a considerable spectrum of gene expression profiles and microbial behaviors in adherent communities. At this time, though, the understanding of how microenviroments within biofilms impact microbial physiology and gene expression, and how these induced states in turn influence intercellular communication pathways and overall community composition and behavior is not well developed.

**FIGURE 1:**
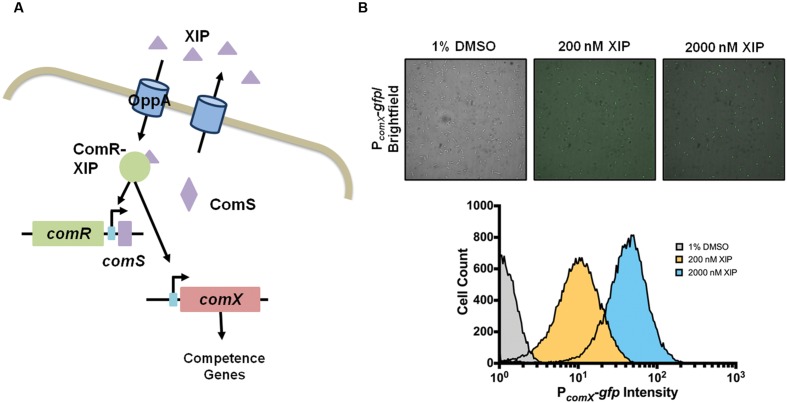
**XIP and the competence circuit.** **(A)** In *S. mutans* genetic competence can be induced by XIP, a peptide that is produced by the cleavage and export of ComS into the extracellular milleu. XIP is imported by the oligopeptide transporter OppA, and once inside the cell binds to a transcriptional regulator, ComR. The ComR-XIP complex binds to early competence genes, *comS* (positive feedback loop) and *comX*. Transcription of *comX* produces an alternative sigma factor, which interacts with RNA polymerase to initiate transcription of the competence regulon. **(B)** In defined medium planktonic phase cultures, the addition of exogenous XIP leads to activation of P*_comX_-gfp* reporter activity in the entire population of cells, as shown by fluorescence microscopy (top) and single-cell flow cytometric analysis (bottom). Data is representative of at least three independent replicates and pictures were taken at x63 magnification.

*Streptococcus mutans* produces at least two secreted peptide pheromones, competence stimulating peptide (CSP; also known as BIP, bacteriocin inducing peptide) and *comX-*inducing peptide (XIP) (Mashburn-Warren et al., 2010). Both of these peptides can stimulate transcription of *comX* (sometimes called *sigX*), which encodes the alternative sigma factor that is the master regulator of late competence genes required for DNA uptake and related cellular processes, e.g., protection of single-stranded DNA and catalysis of homologous recombination. The activation of *comX* (referring to transcription/expression of mRNA) by both of these peptides requires *comRS*. The 17-aa ComS peptide is ribosomally translated, then processed and secreted as XIP by an unknown mechanism. Extracellular XIP is imported by the Opp oligopeptide permease (Mashburn-Warren et al., 2010; Son et al., 2012) and forms a complex with ComR, an Rgg-like transcriptional regulator, that is able to activate *comX* and *comS*; the latter constituting a positive feedback loop (**Figure 1A**) (Mashburn-Warren et al., 2010; Son et al., 2012; Fontaine et al., 2013). ComR-XIP also drives the transcription of the genes immediately downstream of *comS* (SMu.63-68) (Khan et al., 2016). In contrast, the mechanism by which exposure to CSP leads to ComRS-dependent activation of *comX* is not well defined, despite the fact that CSP remains the most intensively studied regulator of genetic competence. CSP is derived from processing and export of the ComC peptide by the ComAB ABC transporter, and is sensed extracellularly by the ComDE two-component system. The primary function of CSP appears to be to activate transcription of a family of bacteriocins (called mutacins in *S. mutans*), consistent with the fact that ComABCDE of *S. mutans* apparently evolved from the BlpABCRH bacteriocin regulatory system found in multiple streptococci (Martin et al., 2006). There is preliminary evidence that treatment of *S. mutans* with CSP may lead to enhanced ComRS production by inducing expression of an endogenous bacteriocin encoded by *cipB* (Perry et al., 2009). There is also evidence of connection between the CSP and XIP signaling systems in that ComX is able to activate *comE* following treatment of *S. mutans* with XIP (Reck et al., 2015; Son et al., 2015b). Relevant here is that, in planktonic cultures, *comX* transcription is only activated by CSP in a sub-population of cells (bimodal response) in medium containing peptides (Lemme et al., 2011). Conversely, *comX* is activated in all cells (unimodally) by XIP in chemically defined medium (**Figure 1B**), but not in complex medium (Son et al., 2012, 2015a); apparently because of interference of Opp-dependent internalization of XIP by peptides.

Bulk phase and microfludic experiments have revealed that environmental factors have a major influence on the competence cascade of *S. mutans*. For example, cells exposed to acidic conditions display reduced *comX* expression in response to XIP and CSP (Guo et al., 2014; Son et al., 2015a), oxygen strongly activates bacteriocin expression (Ahn et al., 2007), and the source of carbohydrate has a substantial influence on activation of *comX* by CSP and on progression of the cells to a competent state (Moye et al., 2016). Here we sought to determine if growth on a surface and in biofilms altered the response of *S. mutans* to XIP. We show that during biofilm maturation activation of *comX* transcription by addition of XIP shifts from a population-wide response to a sub-population response in both wild-type *S. mutans* UA159 and in a hyper-transformable mutant derivative of UA159 (Seaton et al., 2011). We further investigated the origins of phenotypic heterogeneity in biofilms by exploring whether gene products that govern programmed cell death (PCD) and/or growth and survival decisions influence XIP signaling. Using a systematic approach to study the effect of growth in biofilms on the competence cascade is an important step toward understanding how the natural environment impacts horizontal gene transfer and virulence-related traits that are influenced by components of the competence signal cascade.

## Materials and Methods

### Strains and Growth Conditions

*Streptococcus mutans* strains were cultured from single colonies in Brain Heart Infusion (Difco) broth at 37°C in a 5% CO_2_, aerobic atmosphere. For biofilm experiments, strains were grown in the chemically defined medium FMC (Terleckyj et al., 1975) containing a final concentration of 25 mM glucose. Antibiotics were added to growth media in the following concentrations: spectinomycin (spc) 1 mg/mL, kanamycin (kan) 1 mg/mL, and erythromycin (erm) 5 μg/mL.

### Construction of Reporter Gene Fusion Strains

A strain bearing a green fluorescent protein (GFP) reporter gene fusion to the *comX* promoter (P*_comX_-gfp*) was previously constructed (Son et al., 2012). The P*_comX_-gfp* gene fusion is carried on the shuttle vector pDL278 and was introduced into the previously described *S. mutans* strains: *rcrR*-P (kan^r^), Δ*mazF* (kan^r^), Δ*relE* (erm^r^), Δ*mazF*/Δ*relE* (kan^r^ and erm^r^) double mutant, and a Δ*relA* (erm^r^) mutant (Lemos et al., 2005, 2007; Seaton et al., 2011). The GFP used for this fusion is a superfolder GFP that was optimized for brightness in *Staphylococcus aureus* (Lauderdale et al., 2010). Introduction of the P*_comX_-gfp* plasmid into the above strains was performed as follows. Overnight cultures were diluted 1:50 into 200 μL of BHI broth and grown to an OD_600_ = 0.1. At this point, CSP (1 μM) and the P*_comX_-gfp* plasmid (100 ng) were added to the mutant strains. After incubation for 4 h, cultures were serially diluted to 10^-3^ and 100 μL was spread onto BHI agar containing the appropriate antibiotic to select for transformants. All engineered strains were verified by PCR and DNA sequencing.

### Microplate Reporter Assay

Green fluorescent protein promoter activities were assayed using a Synergy microplate reader (BioTek) controlled by Gen5 software. Overnight cultures were washed and resuspended in FMC and then aliquots (10 μL) were added to 200 μL pre-warmed FMC in individual wells of a 96-well plate (black walls, clear bottoms; Greiner Bio-One). Synthetic XIP (amino acid sequence = GLDWWSL; Biomatik) was diluted 100-fold from stock solutions at the concentration tested. XIP was added at the time of inoculation (0 h), or at 5 or 20 h post-inoculation. When added at 5 and 20 h, spent medium was first removed and replaced with fresh FMC, since acidic pH interferes with activation by XIP of *com* gene expression and development of competence (Son et al., 2015a). Sterile mineral oil was gently pipetted onto the cultures in each well and plates were incubated at 37°C. During each experiment, cell growth (OD_600_) and fluorescence were recorded (sensitivity = 65; excitation = 485 nm; emission = 520 nm) at 20 or 30 min intervals. For cell growth, the background OD_600_ of FMC without cells was subtracted from OD_600_ readings. The fluorescence of wild-type or mutant strains without the reporter (+/- XIP) was subtracted from fluorescence readings from P*_comX_-gfp* strains. RFU/OD_600_ values were calculated from 4 wells for each condition. At least three biological replicates were carried out for each experiment.

### Confocal Laser Scanning Microscopy

Overnight cultures were washed and re-suspended in FMC before being diluted 1:20 in fresh medium. Diluted cell suspensions (350 μL) were inoculated into each well of an 8-well μ-Slide (ibidi, USA) chambered coverslip. Synthetic XIP was added at 0, 5, and 20 h after inoculation. When assaying at 5 and 20 h, XIP was added with fresh FMC. Plates were incubated at 37°C in a 5% CO_2_, aerobic atmosphere. Prior to analysis by microscopy, wells were washed three times with PBS and stained with 2.5 μg/mL propidium iodide (PI) for 20 min in the dark at room temperature to assess cell membrane integrity. After removing the stain, wells were washed once more with PBS and then biofilms were kept hydrated with 100 μL of PBS. Biofilm images were acquired using a spinning disk confocal system connected to a Leica DMIRB inverted fluorescence microscope equipped with a Photometrics cascade-cooled EMCCD camera. GFP fluorescence was detected by excitation at 488 nm and emission was collected using a 525 nm (± 25 nm) bandpass filter. Detection of PI fluorescence was performed using a 642-nm excitation laser and a 695-nm (± 53-nm) bandpass filter. All z-sections were collected at 1 μm intervals using an 63X/1.40 oil objective lens. Image acquisition and processing was performed using VoxCell (VisiTech International). Biofilm stacks were also rendered in 3D using Imaris (Bitplane).

### Flow Cytometry

Biofilms were grown in 6-well microtiter plates and dispersed for analysis in a FACSCalibur^TM^ (BD Biosciences) flow cytometer. Specifically, biofilms were grown as indicated above, except that they were cultured in 6-well microtiter plates (Greiner Bio-One) using 2 mL of FMC broth. After 6, 7, and 23 h, biofilms were washed three times with 1 mL sterile PBS before being scraped off and placed in 1.5 mL Eppendorf tubes. PI was used to allow quantification of membrane-compromised cells within biofilm communities. To each sample PI (final concentration = 2.5 μg/mL) was added and cell suspensions were incubated in the dark at room temperature for 20 min. Afterward, cells were washed once with PBS and then 1 μL of cell suspension was placed in 1 mL ice-cold PBS prior to sonication. Cells were sonicated using a Fisher Scientific Model 120 Sonic Dismembrator in the water bath mode at 100% amplitude for 30 s. Although sonicated to produce primarily single cells, doublets and occasionally longer chains could be observed in the suspension (**Supplementary Figure S1**). As a result, forward and side scatter signals were set stringently to allow enumeration of single cells. In total 5 × 10^4^ cells were counted from each event, at a maximum rate of 5 × 10^3^ cells per second, and each experiment was performed in triplicate. Detection of GFP fluorescence was through a 530 nm (± 30 nm) bandpass filter, and PI was detected using a 670-nm long pass filter. Data were acquired for unstained cells and single-color positive controls so that data collection parameters could be properly set. The data were collected using Cell Quest Pro (BD Biosciences) and analyzed with FCS Express 4 (De Novo Software). Graphing and statistical analyses were performed using Prism (GraphPad Software). *x*- and *y*-axis data display logarithmic scales of fluorescence intensity (arbitrary units).

## Results

### Responses of *S. mutans* Cells to XIP during Biofilm Development

**FIGURE 2:**
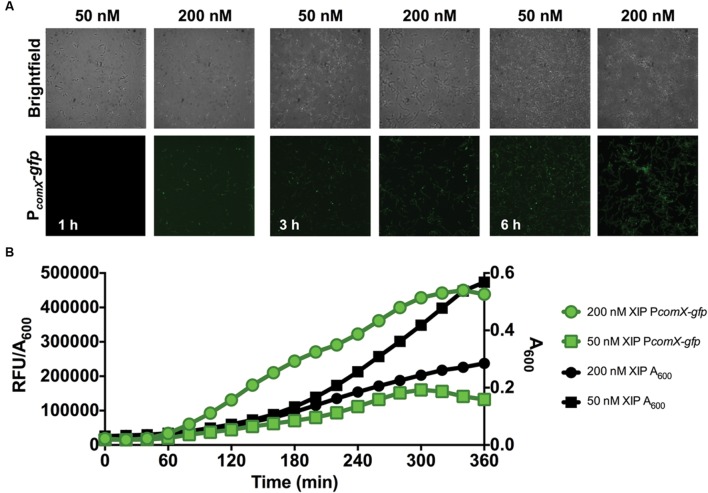
**Time-course analysis of *comX* expression in a 0–6 h biofilm.** **(A)** CLSM images of P*_comX_-gfp* activity in *S. mutans* at the indicated timepoints in an otherwise-wild-type genetic background. Images were obtained at 63X magnification. **(B)** Quantification of P*_comX_-gfp* activity during batch growth in a microtiter-based plate system (see Materials and Methods for details). The data presented plot the fluorescence intensity normalized to the A_600_ of the culture (green lines/symbols). The A_600_ in different conditions is plotted on the secondary *y*-axis in black. Data from both experiments are representative of at least three independent replicates.

Two approaches were employed to monitor *comX* promoter activity during biofilm development: confocal microscopy at defined time points and real-time measurement of total *comX* activity during biofilm maturation in microtiter plates. At all time-points, negative controls that were incubated in the presence of 1% DMSO (without XIP) exhibited no P*_comX_-gfp* activity. As seen in **Figure 2A**, cells became attached to the substratum within 1 h and accumulated over the ensuing 6 h. The expression of *comX*, induced by 50 and 200 nM XIP added at 0 h, was also visualized. GFP expression from the P*_comX_-gfp* promoter fusion was observable within 1 h after induction with 200 nM XIP, whereas GFP fluorescence was only detected after 3 h when 50 nM XIP was added to the growth medium. In the microtiter plate assay (**Figure 2B**), P*_comX_-gfp* activity per cell (RFU/A_600_) increased after 1 h at a linear rate for both the 50 and 200 nM XIP-treated samples, until peak fluorescence was achieved after approximately 5 h of incubation. P*_comX_-gfp* activity per cell was elevated in response to higher concentrations of XIP. As has been reported previously (Wenderska et al., 2012), higher concentrations of XIP had a negative impact on cell growth. In particular, the population incubated with 200 nM XIP had reached an A_600_ of 0.28 after 6 h, compared to an A_600_ of 0.57 for the population cultured in the presence of 50 nM XIP. The untreated control population grew to A_600_ = 0.76.

**FIGURE 3:**
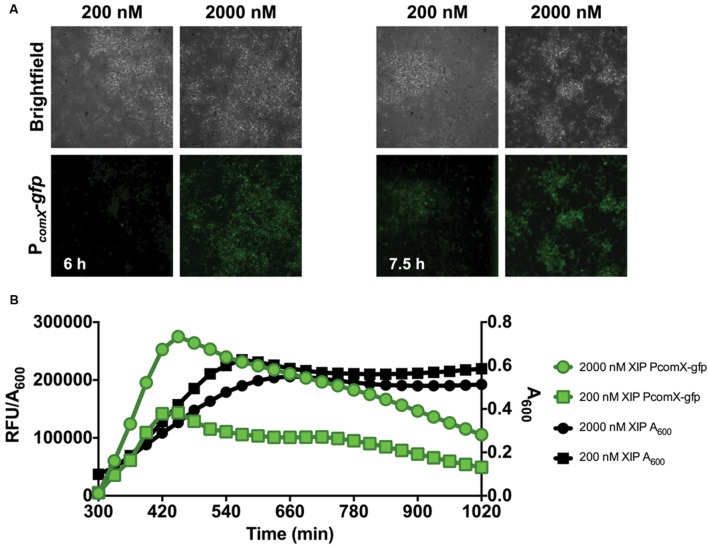
**Time-course analysis of *comX* expression in a 5–7 h biofilm.** **(A)** CLSM images of biofilms of *S. mutans* wild-type carrying a P*_comX_-gfp* at the indicated timepoints (63X magnification). **(B)** Quantification of P*_comX_-gfp* activity, presented as the fluorescence intensity over the A_600_ of the culture (green lines/symbols). The A_600_ in different conditions is plotted on the secondary *y*-axis (black lines/symbols). Data from both experiments are representative of at least 3 independent replicates.

Clearly, cells in the early phases of biofilm formation responded efficiently to XIP. Since cellular behaviors in mature biofilms are distinct in many ways from those is the early stages of biofilm maturation (Beloin and Ghigo, 2005), we also examined how cells responded to XIP in the later stages of biofilm formation. Cells were inoculated in defined medium and allowed to accumulate for 5 or 20 h before XIP was added. At 5 h, 200 nM or 2 μM XIP was added to pre-formed biofilms and GFP levels were monitored by microplate assay and microscopy (**Figure 3**). Higher concentrations of XIP were used because it was empirically determined that the lower concentration used in the early biofilms did not elicit a sufficient P*_comX_-gfp* response. Microscopic analysis showed robust biofilm accumulation in the brightfield channel (**Figure 3A**). P*_comX_-gfp* expression was evident after 1 h of incubation with XIP, although biofilms incubated with 200 nM XIP were more weakly fluorescent at this time point, compared to other conditions. In particular, 200 nM XIP lead to P*_comX_-gfp* activity of 450 × 10^3^ RFU/A_600_ at its peak at the 5 h time point when XIP was added at t_0_, but the same concentration of XIP induced a peak fluorescence of 150 × 10^3^ RFU/A_600_ when added at the 5 h time point (measured at 7 h) (**Figure 3B**). P*_comX_-gfp* activity was produced rapidly after the addition of XIP at 5 h, and increased at a linear rate until peaking at approximately 7 h. Thereafter, no increase in GFP was observed and fluorescence decreased over the subsequent 10 h. Cell accumulation was again adversely affected by XIP, with the control biofilms reaching a final A_600_ of 0.90 compared to an A_600_ of 0.51 for the cell population incubated with 2 μM XIP.

**FIGURE 4:**
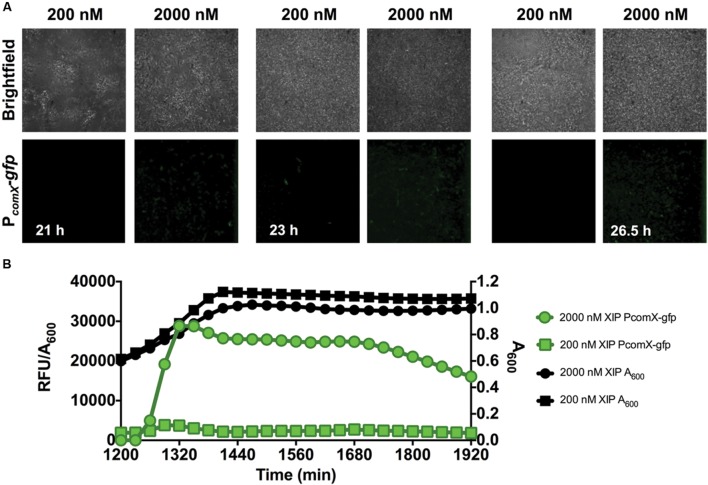
**Time-course analysis of *comX* expression in a 20–23 h biofilm.** **(A)** CLSM images of biofilms showing a strain of *S. mutans* with a wild-type genetic background carrying a P*_comX_-gfp* promoter fusion (63X magnification). **(B)** Quantification of P*_comX_-gfp* activity, presented as the fluorescence intensity over the A_600_ of the culture (green lines/symbols). The A_600_ in different conditions is plotted on the secondary *y*-axis (black). Data from both experiments are representative of at least three independent replicates.

After 20 h of biofilm accumulation without any XIP present, 200 nM or 2 μM XIP was added to pre-formed biofilms and P*_comX_-gfp* activity was visualized with microscopy (**Figure 4A**). Under these conditions, it took approximately 1 h for GFP positive (GFP^+^) cells to be visible when incubated with 2 μM XIP, and 3 h when incubated with 200 nM XIP. There appeared to be less P*_comX_-gfp* activity compared with the earlier time-points and this observation was consistent with what was seen using the microplate assay (**Figure 4B**). Maximal P*_comX_-gfp* activity was approximately 9-fold lower compared to the 5–17 h time points when 2 μM XIP was added to cells. P*_comX_-gfp* activity was near baseline in the microplate assay when 20 h biofilms were incubated in the presence of 200 nM XIP, although some GFP^+^ cells could be observed by microscopy.

### Single-Cell Analysis of Biofilm Populations Reveals a Sub-population Response to XIP

**FIGURE 5:**
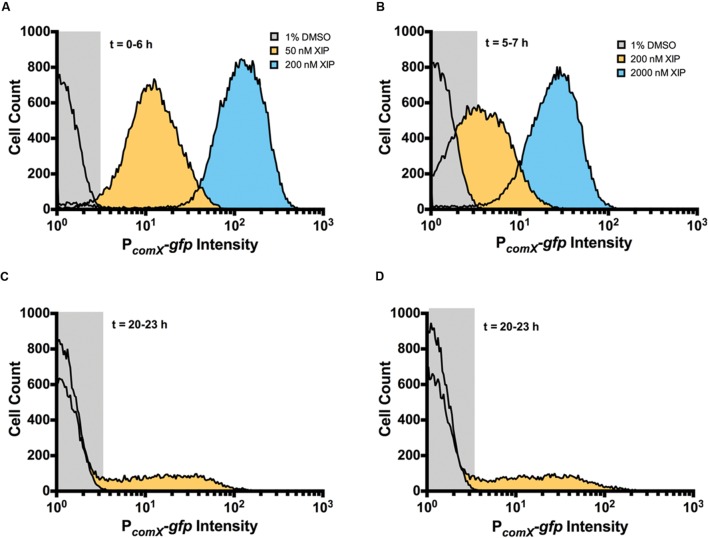
**Single-cell analysis of *comX* expression at differing stages of biofilm development.** Flow cytometry was used to examine GFP intensity and distribution of biofilms that were untreated (gray) or that were induced with XIP (50 nM, orange; 200 nM, blue) at **(A)** 6 h, **(B)** 7 h, and **(C)** 23 h. After incubation with or without XIP, cells were removed from the microtiter plates, sonicated and washed, and then subjected to flow cytometric analysis. **(D)** In this case, biofilms were first removed from the surface of the microtiter plate at 20 h, sonicated and washed, and then incubated with XIP prior to analysis by flow cytometry to determine whether diffusion was limiting exposure to XIP. Data from each experiment is representative of at least three independent replicates.

Cells in planktonic cultures or single cells in a microfluidic system display unimodal responses to XIP, but the microscopic analysis of mature biofilms showed clear evidence of a sub-population (bimodal) response. To quantify the number and intensity of XIP-responsive cells, we conducted a flow cytometric analysis of dispersed biofilm populations. During early biofilm formation, virtually all cells responded to XIP, similar to planktonic cultures (**Figures 1B** and **5A**). Sub-population behaviors in the response to XIP became evident when biofilms were allowed to accumulate for 5 h prior to the addition of XIP. Although the entire population responded to 2 μM XIP at the 7 h time point, only 63% (± 8%) were GFP^+^ when 200 nM XIP was used (**Figure 5B**). Distinct sub-populations were clearly observed at the 20–23 h time point following addition of 2 μM XIP (**Figure 5C**). At the later time point, 10% (± 2%) of the cells were GFP^+^, compared with 91% (± 5%) GFP^+^ cells at 5–7 h in response to 2 μM XIP. To determine if the switch from a population-wide to a sub-population response associated with biofilm maturation could be related to decreased diffusion of XIP through the extracellular matrix, we grew biofilms for 20 h, removed them from the microtiter surface, then washed and sonicated the cells before adding 2 μM XIP. These populations of dispersed biofilm cells responded in a manner similar to the intact biofilms, with 14% (± 7%) of the cells being GFP^+^ (**Figure 5D**) after 23 h. Thus, the change in the response of the population to XIP was associated with the state of the cells and cannot be explained simply by a lack of diffusion of XIP into certain regions of the biofilms. Of course these results do not provide evidence that XIP uniformly penetrates all areas of entire biofilms.

### A Constitutively Hyper-Transformable Mutant Strain Displays a Sub-population Response to XIP in Mature Biofilms

**FIGURE 6:**
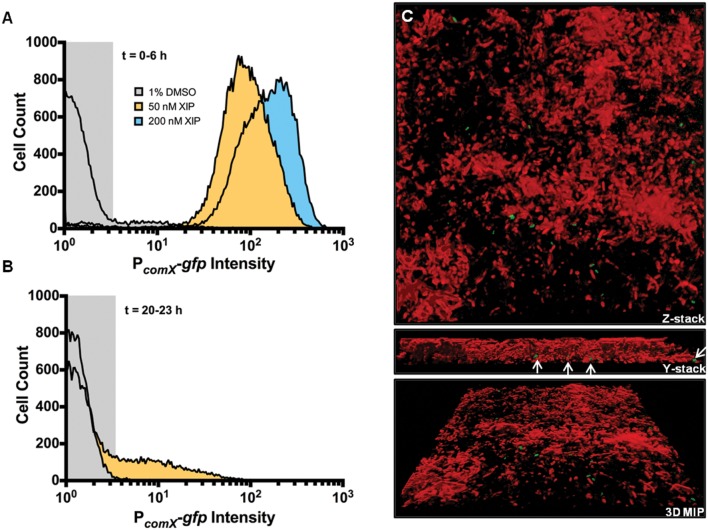
**Effect of XIP on *comX* expression within a biofilm in a hyper-transformable strain of *S. mutans*.** *S. mutans rcrR*-P carrying the P*_comX_-gfp* fusion was cultured in biofilms, then processed and analyzed by flow cytometry as above. **(A)** Cell profiles from early biofilms (0–6 h). **(B)** Cells from 20 to 23 h biofilms. Histograms represent the distributions of GFP^+^ cells in the total population that was recorded (5 × 10^4^ cells). **(C)** Three-dimensional reconstruction of a 23 h *rcrR*-P P*_comX_-gfp* biofilm not treated with XIP (1% DMSO). P*_comX_-gfp* positive cells (green) and cells not expressing GFP (red) are shown. Cells not responsive to XIP were stained with SYTO 60 (2 μM) (Thermo Fisher Scientific), a cell permanent dye, for 20 min in the dark at room temperature. SYTO 60 was excited using a 642-nm excitation laser and the emission was collected using a 695-nm (± 53-nm) bandpass filter. White arrows show the location of GFP^+^ cells on the surface of the glass coverslip, or in the deeper layers of the biofilm. 3D renderings are representative of at least three independent replicates and images were collected 63X magnification.

Replacement of the *rcrR* gene of *S. mutans* UA159 with a polar antibiotic resistance marker (strain designation *rcrR*-P) results in a 10^4^-fold increase in transformation efficiency in cells that are not treated with XIP or CSP, compared to the wild-type strain (Seaton et al., 2011). This competence phenotype has been associated with multiple changes in gene expression associated with the *rcrR-P* mutation that include loss of RcrR binding to the promoter of *comX*, changes in (p)ppGpp levels, and altered expression of *rcrP*, *rcrQ*, and two peptides encoded in the 3′ end of *rcrQ* (Seaton et al., 2011, 2014; Ahn et al., 2014). Importantly, the *rcrR-P* strain is hyper-sensitive to XIP, with the mutant showing marked growth inhibition in concentrations of XIP that have a comparatively small effect on the wild-type strain. Flow cytometric analysis revealed that the *rcrR-P* strain responded robustly to XIP during initial biofilm accumulation (**Figure 6A**), with P*_comX_-gfp* intensity being significantly higher than that of the wild-type strain after addition of 50 or 200 nM XIP. Interestingly, *rcrR*-P exhibited bimodal GFP expression without addition of XIP, both in planktonic and biofilm cultures (**Figure 6A**; **Supplementary Figure S2**). Spontaneous P*_comX_-gfp* activity was observed between the 0 and 6 h time points, with 5% ± 1% of the cells producing green fluorescence without the addition of XIP. Similarly, the elevated P*_comX_-gfp* activity displayed by the *rcrR*-P strain compared to the strain with GFP levels in the wild-type genetic background in the early biofilms was observed in the 20–23 h samples when 2 μM XIP was added to biofilms (*rcrR*-P 16% ± 2%) (**Figure 6B**). At 20–23 h, we also observed GFP^+^ cells in biofilms that were not exposed to exogenous XIP in the *rcrR*-P mutant genetic background (**Figure 6C**). Notably, GFP^+^ cells were localized near the substratum. When analyzed by flow cytometry, 20–23 h biofilms were not particularly rich in GFP*^+^* cells; only 0.9% ± 0.3% of the total cells was GFP*^+^*.

### Visualization of Live Cells That Do Not Respond to XIP

**FIGURE 7:**
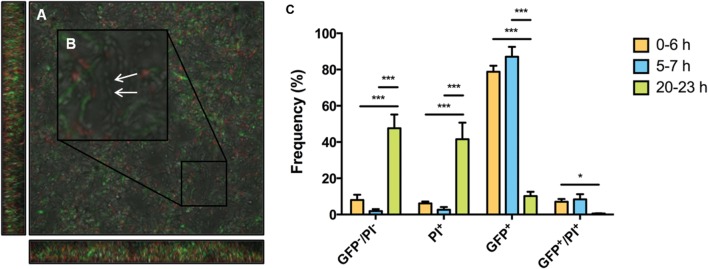
**Microscopy and quadrant analysis of *S. mutans* biofilm with P*_comX_-gfp* reporter and propidium iodide (PI) staining.** At 23 h, *S. mutans* wild-type carrying a P*_comX_-gfp* reporter were visualized after the addition of 2 μM XIP for 3 h. Cells were also simultaneously stained with PI to discriminate between live cells and those with compromised membranes (PI^+^). Images were obtained at 63X magnification. **(A)** GFP, PI, brightfield channels were merged. **(B)** An area was selected and increased in size to allow the discrimination of different cell phenotypes, including live cells that were not responding to XIP (white arrow). **(C)** Biofilm populations at selected time points sorted into four distinct phenotypes by flow cytometry. The four quadrants represent the percentage (of total cells counted) GFP positive cells, PI positive cells, GFP and PI positive cells and GFP and PI negative cells. At 0–6 h 200 nM XIP was used to induce P*_comX_-gfp* activity, whereas 2 μM XIP was used at the 5–7 h and 20–23 h time points. The statistical significance of differences between time points in distinct sub-populations was calculated using two-sample (unpaired) *t*-tests (^∗^*P* ≤ 0.05; ^∗∗∗^*P* ≤ 0.001).

Maturation of biofilms leads to a transition from a population-wide to a sub-population response of *S. mutans* to XIP. However, it is likely that the biofilms contain dead cells. To determine if the sub-population distribution of GFP^+^ cells at the 20–23 h timepoint was simply due to the fact that there was a large proportion of dead cells that would be unable to respond to XIP, and/or cells were killed as a result of exposure to high concentrations of XIP, we used flow cytometric quadrant analysis and CLSM to visualize PI staining and P*_comX_-gfp* reporter expression. Microscopy at 20–23 h showed cells that were GFP^+^, PI positive (PI^+^, i.e., dead or damaged) and GFP^-^/PI negative (PI^-^, intact but non-responsive to XIP). There was no obvious spatial arrangement of live or dead cells, or responders or non-responders, although all dead cells were non-responders (**Figure 7**). Live cells that were not responding to XIP at the time-point tested were visible during microscopy (**Figure 7B**). Quantification of cell phenotype distributions using quadrant analysis showed that 48% ± 8% of biofilm cells at 20–23 h were GFP^-^/PI^-^ (**Figure 7C**). This contrasts with the earlier time points where only a small percentage of cells were non-responsive at 0–6 h or 5–7 h, with 8% ± 2% and 2% ± 1% of the populations being non-responsive, respectively. As observed above, the percentage of GFP^+^ cells within the 20–23 h biofilm was significantly decreased compared to earlier time points, with 10% ± 2% GFP^+^ cells at the later time point, versus 87% ± 4% at 5–7 h and 79% ± 3% at 0–6 h. Another measure of the decreased response of 20–23 h biofilms to XIP was the significant decrease in GFP^+^/PI^+^ cells in this population compared to 0–6 h (0–6 h, 7% ± 1%; 20–23 h, 0.6% ± 0.1%). The simplest interpretation of these data is that the permeability to PI reflects that the cells are dead or in the process of lyzing, but the presence of detectable P_comX_-*gfp* activity indicates that the cells had produced GFP at some point during the maturation of the biofilm.

### Responses to XIP in Biofilms Are Regulated by Growth/Survival Modulators

**FIGURE 8:**
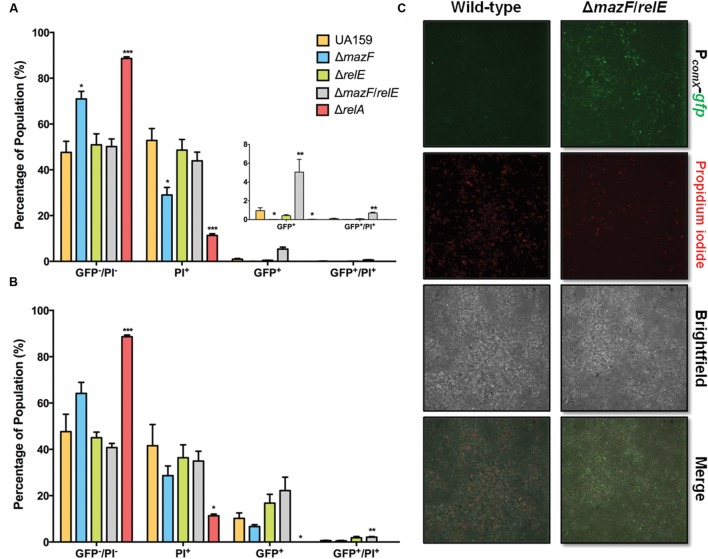
**Effect of Δ*mazF* and Δ*relE* mutations on *comX* activation in a mature biofilm.** Flow cytometry was used to calculate the percentage of cells in each sub-population of mature biofilms at 23 h. 200 nM XIP **(A)** or 2 μM XIP **(B)** was added to wild-type, Δ*mazF*, Δ*relE*, and Δ*mazF*/*relE* biofilms at 20 h and incubated for 3 h. The sub-populations represent GFP positive cells, PI positive cells, GFP and PI positive cells, and GFP and PI negative cells; calculated as the percentage (of total cells counted) in each sub-population. The statistical significance of differences between wild-type and mutant strains in distinct sub-populations was calculated using two-sample (unpaired) *t*-tests (^∗^*P* ≤ 0.05; ^∗∗^*P* ≤ 0.01; ^∗∗∗^*P* ≤ 0.001). **(C)** Confocal laser scanning microscopy of wild-type and Δ*mazF*/*relE* biofilms after the addition of 200 nM XIP for 3 h, starting at hour 20. Biofilms were stained with PI, along with imaging of P*_comX_-gfp* activity. Images are representative of three independent experiments and were taken at 63X magnification.

We sought to explore the molecular basis for a change in response to XIP as biofilms mature. Type II toxin-antitoxin systems and the (p)ppGpp synthetase/hydrolase, RelA (sometimes called Rel), have been implicated in PCD and growth arrest in multiple bacteria, including *S. mutans* (Christensen et al., 2001; Engelberg-Kulka et al., 2005; Lemos et al., 2005; Nascimento et al., 2008; Kolodkin-Gal et al., 2009; Maisonneuve and Gerdes, 2014). We hypothesized that changes in the percentage of dead cells, and/or relaxation of growth arrest, may increase the responsiveness of cells to XIP. We explored this hypothesis using flow cytometric quadrant analysis and CLSM, analyzing the behaviors of strains lacking the Type II toxins MazF or RelE, a Δ*mazF*/*relE* double mutant, and a Δ*relA* mutant (**Figure 8**). Of note, RelA in *S. mutans* is one of three (p)ppGpp synthetases, the other two are RelP and RelQ, with RelP producing the majority of (p)ppGpp during exponential growth (Lemos et al., 2007). However, RelA also possess (p)ppGpp hydrolyze activity, and deletion of RelA therefore leads to increased basal levels of (p)ppGpp during exponential growth (Nascimento et al., 2008). The addition of 200 nM XIP resulted in P*_comX_-gfp* activity in 1.0% ± 0.3% of wild-type cells at the 20–23 h time-point. However, in the Δ*mazF*/*relE* double knockout mutant we observed a five-fold increase in the number of GFP^+^ cells (5% ± 1%; *p* = 0.008). The GFP^+^/PI^+^ population was also increased in the Δ*mazF*/*relE* mutant compared to the wild-type strain (wild-type, 0.1% ± 0.1; Δ*mazF*/*relE*, 0.6% ± 0.1). The increased number of GFP^+^ cells in the Δ*mazF*/*relE* mutant was clearly evident when biofilms were examined by CLSM (**Figure 8C**). Of note, the proportions of PI^+^ cells were not significantly different between the biofilms formed by wild-type and the Δ*mazF*/*relE* mutant strains when measured by flow cytometry (wild-type, 53% ± 5%; Δ*mazF*/*relE*, 44% ± 4%; *p* = 0.24). However, by microscopy there were fewer PI^+^ cells visible in the Δ*mazF/relE* mutant biofilms at 23 h compared to the wild-type biofilms at the same time point. When 2 μM XIP was added at the 20 h time point, there was a two-fold increase in GFP^+^ cells in the Δ*mazF*/*relE* mutant, although this was not significant (wild-type, 10% ± 3%; Δ*mazF*/*relE*, 22% ± 6%; *p* = 0.13). The GFP^+^/PI^+^ Δ*mazF*/*relE* population was significantly greater (four-fold; *p* = 0.004) than in the biofilms formed by the wild-type strain (wild-type, 0.6% ± 0.1; Δ*mazF*/*relE*, 2.1% ± 0.2). Loss of Δ*relA* greatly reduced the percentage of cells stained with PI in the sample treated with 200 nM XIP (11% ± 1%) or 2 μM XIP (10% ± 1%), but also resulted in a significantly lower proportion of GFP^+^ cells (200 nM XIP, 0.02% ± 0.01; 2 μM XIP, 3% ± 0.3%). The Δ*mazF* single mutant also displayed reduced cell death when incubated in the presence of 200 nM XIP (PI^+^, 29% ± 3%; GFP^+^, 0.4% ± 0.1%).

## Discussion

Previous work has explored the impact of growth phase, environmental conditions (e.g., pH), and media composition on *comX* expression in response to signal peptides in planktonic cultures. These studies highlighted that fluctuations in environmental conditions created by the formation of microenvironments in biofilms could, therefore, substantially modify responses to the signaling molecules governing competence and virulence traits of *S. mutans* that have been shown to be under the control of the competence regulon. Here, we begin to shed light on how the influence of the XIP molecule on cellular behaviors is modified by biofilm growth. The results presented demonstrate that cells that were adhering and accumulating on a substratum, two essential activities in the early phases of biofilm formation, responded in a population-wide manner to the addition of XIP (**Figure 1B**), essentially similar to what has been described for planktonic cells that were cultured batch-wise or as adherent cells in a low cell density environment in microfluidic studies (Son et al., 2012, 2015a). In contrast to planktonic and low-density adherent populations, establishment of *S. mutans* in mature, naturally formed biofilms at higher cell densities lead to only a small sub-population of cells activating *comX* when exposed to exogenously supplied XIP. The differences between the early biofilms and the mature biofilms were striking, as not only was a smaller percentage of cells producing GFP at the later time-point, but an increased amount of XIP was also required to induce *comX* expression.

Interestingly, the CSP-ComDE pathway for activation of *comX* leads to bimodal P*_comX_-gfp* activation in both planktonic (Lemme et al., 2011) and biofilm growth (Aspiras et al., 2004) modes. Indeed, *comX* activation by CSP appears similar to the Agr peptide-mediated autoinduction cascade in the Gram-positive pathogen *Listeria monocytogenes*, which exhibits bimodal behavior in planktonic and biofilm growth modes (Garmyn et al., 2011). In contrast, the proportion of cells activating *comX* in response to XIP decreases substantially as cell density increases. A similar phenomenon has been observed in batch-cultured planktonic cells, where natural transformation rates in *S. mutans* decline as cell density increases (Dufour et al., 2015), although this decrease was shown to be, at least in part, due to low pH having an adverse influence on peptide-dependent activation of the competence cascade (Son et al., 2015a). However, other factors clearly impact competence in response to cell density in *S. mutans.* For example, the *hdrRM* operon, a two gene regulatory system that is a distal regulator of competence, is expressed in high density cultures of *S. mutans* with loss of *hdrM* having a positive effect on transformation efficiencies (Merritt et al., 2007; Okinaga et al., 2010); although, to our knowledge, the effects of HdrRM have yet to be assessed in model that utilizes naturally formed biofilms. Importantly, the response of cells that were established in mature biofilms, but that were subsequently challenged with XIP following dispersal was the same as the cells in intact biofilms (**Figure 5**). Clearly, then, neither high cell density or lack of XIP diffusion through the extracellular matrix or through dense aggregates of cells can explain the different behaviors of early and mature biofilm cells, or early exponential phase planktonic cells and mature biofilms. More likely, heterogeneity within microenviroments in the biofilms modified gene expression patterns resulting in altered physiologic states of individual cells that blunt their ability to sense and/or transduce the XIP signal into changes in *comX* promoter activity.

In order to obtain more detailed insights into the switch from a population-wide to a sub-population response, we explored P*_comX_-gfp* activation in the hyper-transformable strain *rcrR*-P. In particular, it is known that *comX* expression in *rcrR*-P is constitutively elevated in cells growing in planktonic culture (Seaton et al., 2011; Kaspar et al., 2015), and we set out to discover if this was also true in mature biofilms. Somewhat surprisingly, the *rcrR*-P mutant exhibited bimodal *comX* activation without addition of exogenous XIP, in early and mature biofilms, although the proportion of GFP^+^ cells in mature biofilms decreased substantially, and those cells that were activated tended to be located close to the substratum. The observation that *comX* activation is reduced in *rcrR*-P mature biofilms (with or without XIP addition), similar to wild-type, further implies that growth on a surface and possibly certain microenvironments create conditions where cells either cannot properly receive the XIP signal or are actively blocking genetic competence at the level of ComR-XIP activation of *comX*. Spatial heterogeneity in biofilms, that is, gradients of nutrients, pH, oxygen, signaling molecules, and many other substances, create differences in gene expression profiles (Stewart and Franklin, 2008). Notably, low pH has been shown to be a major block on *comX* expression in planktonic cells (Son et al., 2015a), while also appearing to have a heterogenous spatial distribution in mixed-species oral biofilms, including *S. mutans* (Xiao et al., 2012). A more detailed analysis of the impact of physiological heterogeneity on *comX* expression is warranted. However, studying *comX* activation in the *rcrR*-P strain has provided further evidence that there are substantial differences in the response of planktonic and biofilm cells to XIP, likely imposed by exogenous inputs present in biofilm populations.

Interestingly, and a novel dimension to the control of genetic competence in *S. mutans*, mutations in the genes for the MazF and RelE type-II toxins modified the subpopulation response of *S. mutans* biofilms to XIP. Type-II toxins have been most intensively studied using *Escherichia coli*, and MazF of *E. coli* has been implicated in promoting PCD by stressed cells (Engelberg-Kulka et al., 2005; Kolodkin-Gal et al., 2009) and inducing a reversible state of bacteriostasis (Zhang et al., 2003; Mok et al., 2015). By contrast, RelE, is activated during amino acid starvation and leads to reduced translation (Christensen et al., 2001; Pedersen et al., 2003). We observed that deletion of *mazF* alone decreased cell death in a mature biofilm in cells treated with 200 nM XIP, whereas loss of RelE did not, perhaps indicative of a conserved role for MazF in *E. coli* and *S. mutans*. Interestingly, it was only after *mazF* and *relE* were both disrupted that a noticeable increase in the percentage of cells responding to XIP was observed. It is noteworthy that Lemos et al. (2005) also found that deletion of both MazF and RelE in *S. mutans* leads to more pronounced phenotypes, such as acid tolerance within biofilms, than single mutations. Syed et al. (2011) have shown that *S. mutans* MazF is a toxic protein with RNase activity that most likely contributes to growth arrest and dormancy during as-yet-to-be-defined conditions. Collectively, our data suggest that factors involved in growth arrest, or cell death, are leading to a decrease in the proportion of the population that is capable of XIP-dependent activation of *comX*. Thus, we propose that abolishment of both MazF and RelE leads to a larger population of cells that are alive (via MazF), while diminishing the proportion of cells that enter a state of bacteriostasis (via MazF and RelE) in a way that results in increases in the proportion of cells and intensity of the response to XIP, as measured by increased GFP expression from the *comX* promoter.

Here, we also show that (p)ppGpp levels can modulate the levels of *comX* activation in a biofilm population. *S. mutans* produces three enzymes that govern (p)ppGpp production: RelA, with both synthase and hydrolase activity, and two enzymes, RelP and RelQ that appear to have only synthetase activity (Lemos et al., 2007; Nascimento et al., 2008). In planktonic *S. mutans* cells, (p)ppGpp metabolism has been linked to genetic competence via the *rcrRPQ* operon (Seaton et al., 2011, 2014) and more recently by exploring the competence phenotypes in Δ*relA* and (p)ppGpp^0^ strains (Kaspar and Burne, in preparation). In the planktonic phase RelA deletion also leads to reduced *comX* expression (Kaspar and Burne, in preparation), although the mechanism by which (p)ppGpp levels affect *comX* promoter activity is not yet established. Based on results presented herein, a model that includes toxin-antitoxin modules could be proposed. Specifically, it was observed that (p)ppGpp levels in *E. coli* populations vary stochastically, with high levels leading to induction of toxin-antitoxin loci (Maisonneuve et al., 2013). Our data suggest that increased levels of MazF and RelE would have a negative impact on *comX* activation within biofilms. This leads to the hypothesis that the increased levels of (p)ppGpp that would occur from loss of the RelA hydrolase activity could enhance toxin-antitoxin levels, thereby modulate the response of the population to the XIP molecule via T/A MazF and RelE activities. We are currently exploring other factors, such as the RcrRPQ pathway and Clp proteases that modulate T/A stability, as contributors to the competence phenotypes in biofilms.

In summary, we used single-cell analysis and a reporter system to demonstrate that wild-type *S. mutans* decreases XIP-dependent activation of *comX* as biofilm biomass increases. It is also important that a strain that expresses constitutively high levels of *comX* and is highly transformable in planktonic cultures displays the same general trend in both the presence and absence of XIP. While pH has been shown to be a dominant factor in XIP signal transduction (Son et al., 2015a), and other factors strongly influence XIP-dependent activation of *comX*, this study is the first to show that bistable responses to XIP can be induced by biofilm growth and that the XIP-dependent activation of *comX* may be controlled in part by Type II toxins and (p)ppGpp; which influence both the proportion of cells that can respond and intensity of the response in individuals cells. The data with dispersed biofilms also supports that biofilm growth is sufficient to induce a state where XIP-dependent activation of a sub-population will not occur in a large segment of the population. Thus, a biofilm is a clearly distinct developmental state from planktonic cultures, allowing gradients to confer phenotypic heterogeneities and altered responses to signaling molecules. Closer inspection of biofilm microanatomy and spatial/temporal heterogeneity, and their associated impacts on signaling pathways is ongoing.

## Author Contributions

RS contributed to conception, design, acquisition, analysis, and interpretation, drafted, and critically revised the manuscript; RB contributed to conception, design, and interpretation, drafted, and critically revised the manuscript. Both authors gave final approval and agree to be accountable for all aspects of the work.

## Conflict of Interest Statement

The authors declare that the research was conducted in the absence of any commercial or financial relationships that could be construed as a potential conflict of interest.
