# Supplementary material for: Growth of Streptococcus mutans in Biofilms Alters Peptide Signaling at the Sub-population Level
Source: Front Microbiol. 2016 Jul 15;7:1075. doi: 10.3389/fmicb.2016.01075 (PMC4946182; doi:10.3389/fmicb.2016.01075)
Supplement: Supplementary file 1 [file Presentation_1.PDF]

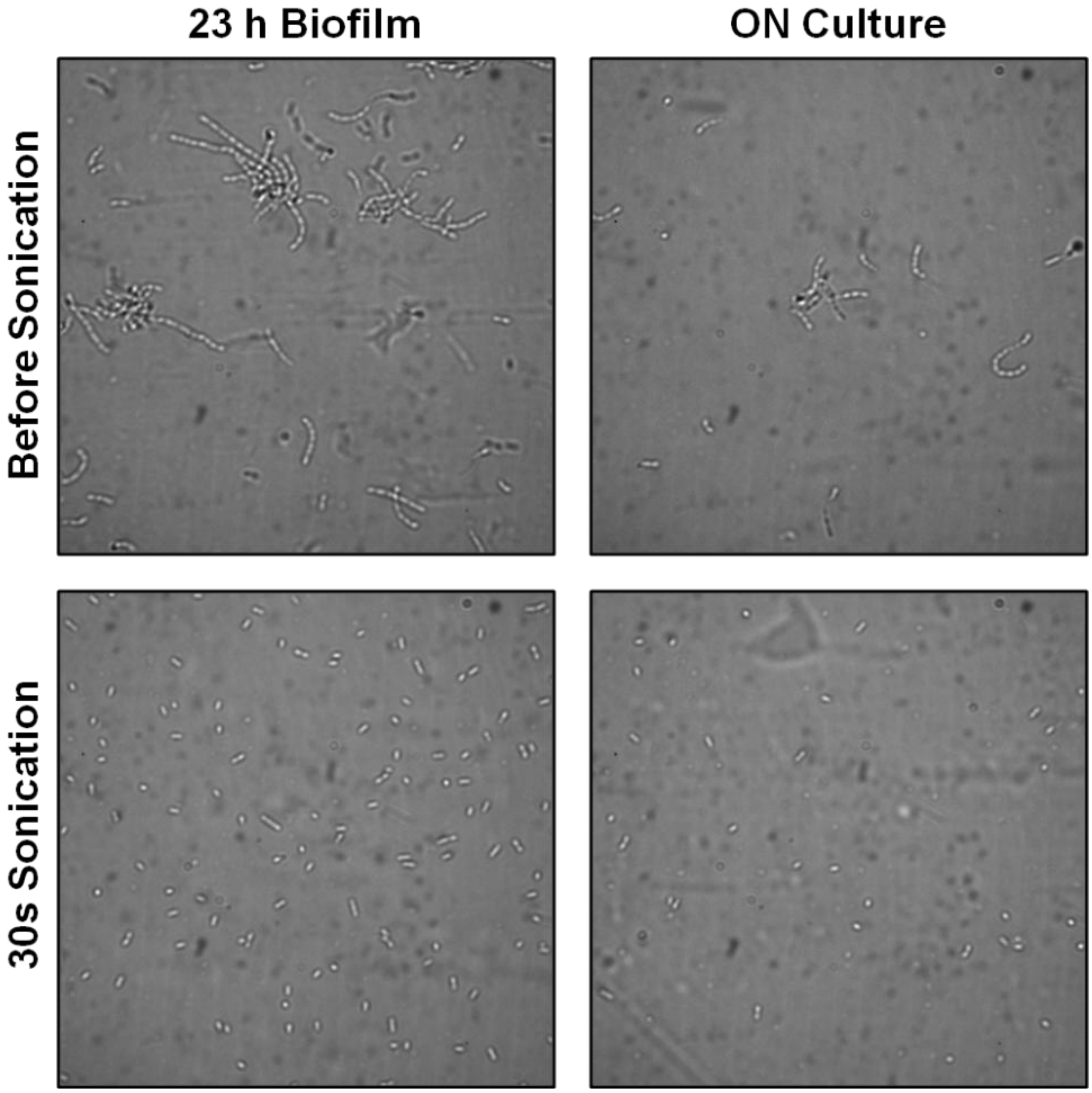

S1. Sonication of *Streptococcus mutans* cultures to single cells. Cells were sonicated using a Fisher Scientific Model 120 Sonic Dismembrator in the water bath mode at 100% amplitude for 30 s. Afterwards 10 µL of the sonicated sample was placed on a microscope slide and covered with a coverslip for microscopic analysis. For both the overnight (ON) culture and 23 h biofilm streptococci chains were disrupted to produce single, and doublet cells.

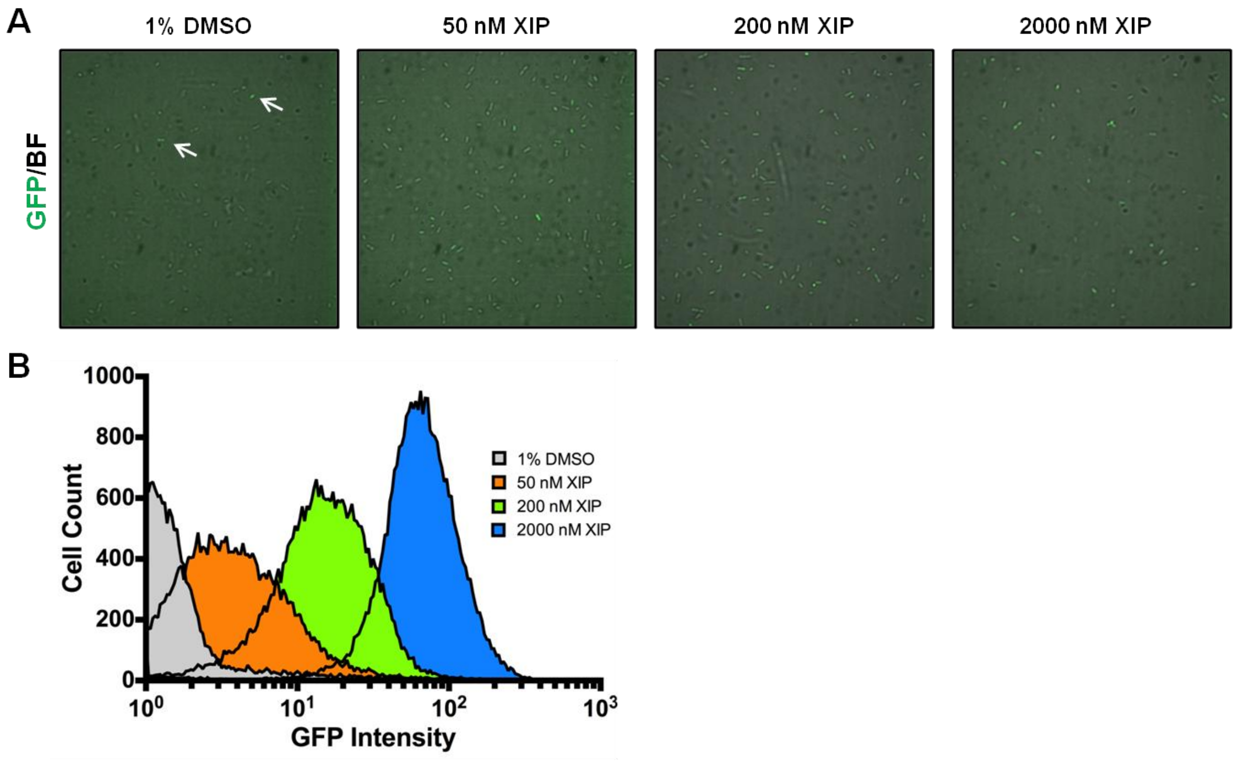

S2. Single cell analysis of *comX* expression in the hyper-transformable strain *rcrR*-P cultured planktonically. *S. mutans rcrR*-P was cultured in defined medium for a period of 6 h, in the presence or absence of exogenous XIP and (A) P_{comX}-*gfp* activity was visualized using confocal microscopy. Green fluorescent cells were observed in the sample not containing exogenous XIP (cells highlighted by white arrows). (B) Flow cytometric analysis also confirmed the presence of *comX* expressing cells in the control samples that were untreated (grey), as well as those incubated with exogenous XIP (50 nM, orange; 200 nM, green; 2 µM, blue).
